# Mobile nutrition and health management platform for perioperative recovery: an interdisciplinary research achievement using WeChat Applet

**DOI:** 10.3389/fmed.2023.1201866

**Published:** 2023-05-24

**Authors:** YuJia Wu, Xin Wang, Feng Gao, JinRong Liao, Jie Zeng, Lin Fan

**Affiliations:** ^1^Department of Anesthesiology, Stomatological Hospital of Chongqing Medical University, Chongqing, China; ^2^Chongqing Key Laboratory of Oral Diseases and Biometal Sciences, Chongqing, China; ^3^Chongqing Municipal Key Laboratory of Oral Biomedical Engineering of Higher Education, Chongqing, China; ^4^Department of Anesthesiology, The Sixth People’s Hospital of Chongqing, Chongqing, China

**Keywords:** mobile applications, WeChat mini-program, nutrition and health management, remote medical recovery, perioperative

## Abstract

**Background:**

In recent years, the number of people using mobile applications to promote health and welfare has exponentially increased. However, there are fewer applications in the field of ERAS. How to promote the rapid rehabilitation of patients with malignant tumor surgery during perioperative period and the mastery of its long-term nutritional state is a problem to be solved.

**Objective:**

The purpose of this study is to design and develop a mobile application, and use Internet technology to better manage nutritional health to achieve rapid recovery of patients with malignant tumor surgery.

**Methods:**

This study is divided into three stages: (1) Design: use participating design to make the MHEALTH APP adapt to the clinical practice of nutritional health management; (2) Development: the WeChat Applet of Nutrition and Health Assessment (WANHA) developed using the Internet technology development, and web management programs. (3) Procedure test: patients and medical staff evaluate WANHA’s quality (UMARS), availability (SUS), and satisfaction, and conduct semi-structured interviews.

**Results:**

In this study, 192 patients with malignant tumor surgery, 20 medical staff used WANHA. Patients with nutritional risks are supported by supporting treatment. The results show that patients who have not been treated during the perioperative period, the incidence of postoperative complications (22.4%) and the average hospitalization time after surgery decreased significantly. The incidence of nutritional risks is nearly more than the preoperative level. 45 patients and 20 medical staff participated in the survey of WANHA’s SUS, UMARS, and satisfaction. In the interview, most patients and medical personnel believe that the procedure can improve the current medical services and nutritional health knowledge levels, promote the communication of medical staff and patients, and strengthen the nutritional health management of patients with malignant tumors under the concept of ERAS.

**Conclusion:**

WeChat Applet of Nutrition and Health Assessment is a MHEALTH APP that enhances the nutrition and health management of patients with perioperative period. It can play a huge role in improving medical services, increasing patient satisfaction, and ERAS.

## 1. Introduction

Enhanced Recovery After Surgery (ERAS) is a comprehensive approach to perioperative care. This concept was first introduced in colon surgery (1), but has since been applied to a variety of surgical procedures. ERAS has been a significant development in modern surgery in recent years. In its core, ERAS is based on evidence-based practices in a variety of fields, including surgery, anesthesia, nutrition, and more, in order to optimize the physical and mental well-being and organ function of patients before surgery (2, 3). This helps to reduce the stress response during surgery and the incidence of postoperative complications, leading to faster postoperative recovery.

Nutritional therapy is an important factor. Nutrition intake during perioperative surgery is associated with postoperative complications and increased mortality (4, 5). A study by ChunHua et al. (6). On nutritional therapy for patients with common malignant tumors in China revealed that 68.78% of patients did not receive any nutritional therapy (7, 8). Additionally, severe malnutrition in colorectal cancer (PG-SGA score ≥ 4 points) was found in 61.5% of colorectal cancer patients, which is higher than the rates reported in other countries. This suggests that the issue of inadequate nutritional treatment for malignant tumor patients in China is a major obstacle to the success of ERAS. At this stage, the management model under the ERAS concept optimizes the perioperative management process; however, it lacks tracking and evaluation of the patients’ long-term nutritional status. As this could potentially impact the patient’s overall treatment experience and quality of life in terms of both psychology and physiology, therefore to fully optimize recovery and improve the patient experience, it may be beneficial to consider incorporating long-term nutritional monitoring into the ERAS model.

With the advent of the “Internet+” the barriers of time and space have been eliminated, allowing for the easy access to information at any location and time. Therefore, mobile health management has gained increasing attention (9). Mobile health applications (MHEALTH APP) are a key component of mobile health management. These are software programs that utilize smartphones, tablets, and other mobile devices to improve health outcomes (10). Owing to their low cost, convenience, and speed, personalized services are conducive to the effectiveness of limited medical service resources (11). Currently, there are a wide range of medical applications in China and abroad, such as the mobile medical plan Pro (MPP), KRAFTVARKET, and Imanage (12–14). The global digital report in 2022 shows that there are 5.31 billion mobile phone users and 4.95 billion Internet users globally. Among them, China has 1.630 billion mobile phone users and 1.02 billion Internet users, which guarantees traffic for the distribution and promotion of the MHEALTH APP. Compared with the traditional MHEALTH APP development costs, long cycles, tedious use, low patient satisfaction, and decreased service efficiency of mobile medical care (15), WeChat that is a social software similar to Twitter and Facebook, but it has developed more health functions, applets can achieve geometric social networks relying on WeChat applications with monthly living resources of over 1 billion. It has the characteristics of simple development, convenient use, and strong service targeting. This can effectively solve the difficulties faced by patients and enhance the service capabilities of mobile medical care.

The WeChat Applet of Nutrition and Health Assessment (WANHA) was developed by the Department of Anesthesiology at the Department of Oral Hospital of Chongqing Medical University in 2021. It has successfully developed WeChat mini-programs for dental phobia and has promoted clinical practice (16). WANHA is a long-range and intelligent medical assistance management platform program that can provide perioperative and long-term nutritional health assessment, guidance, and tracking, making it more convenient and effective for patient management. The WeChat Mini-Program can help physicians manage high levels of information and reduce the burden of repetitive tasks in treatment evaluation. This leads to a lower likelihood of errors and omissions.

This study aimed to develop WANHA and investigate how to evaluate WANHA after use, and to test and verify whether the mini-program can play a useful role in the perioperative period and long-term management of patients with malignant tumors.

## 2. Methods

### 2.1. Research design

This study uses participatory design methods (17), which involve the active participation of a range of stakeholders including patients, anesthesiologists, surgeons, and computer scientists. These stakeholders are able to contribute to the design process by identifying issues and pain points, proposing solutions, and evaluating results. This design method is based on the ethical principle that all users involved in the design process have the right to voice their opinions and contribute to the subjective initiative of the group (18, 19). This ensures that the design results meet the needs and usefulness of all parties in the design process. The goal of this approach was to make WANHA more appealing to all users. As needed, additional face-to-face and online meetings were held during the study period.

### 2.2. Research tools

This study involves the use of four research tools: Nutritional Risk Screening 2002 (NRS2002), system availability tables (System USABILITY Scale, SUS), mobile app quality rating table (UMARS), and semi-structural interview survey.

Nutritional risk screening (NRS2002) is based on evidence-based medicine. After verified screening tools, nutritional risks can be quickly and comprehensively discovered, providing scientific basis for clinical nutritional support treatment (5A, Class A recommendation), and are widely extensive recommended for patients after admission to the hospital (20).

This table includes three parts: damage to nutritional conditions (BMI, decreased weight, reduction of eating, etc.), severity of the disease (whether it is tumor patient, no diabetes, acute seizures of chronic diseases, etc.), whether age ≥ 70 years (if 70 years of age; if you are Age ≥ 70 years old, add one point). The total score of NRS2002 is 0–7 points, <3 points are not nutritional risk, and ≥ 3 points are nutritional risks. Total score ≥ 3 points: Patients are at risk of nutrition, need nutritional support, and formulate nutritional treatment plans in combination with clinical practice. Total score < 3 points: review nutritional risk screening every week. NRS2002 is by far the only nutritional risk screening tool with whether the clinical ending improves. The short table is entered into the WANHA patient terminal, and the scoring standard is entered at the same time to facilitate the program direct score.

The system available meter (SUS) is a standardized and quantitative availability table, which uses a fast and effective method to collect the usability of the system (21). This scale includes five positive and five negative problems. Li Kett five-point scoring method is used: 1 = Very disagreement, 5 = Very agreed, and the score is from 0 to 4 points. The contribution of the odd number problem is that the position of the scale is reduced by 1, and the score contribution of the even number of problems is 5 to minus the position of the scale, multiply the sum of the score at 2.5, and get the total value of the SUS to 0–100.

The mobile application quality rating table (UMARS) is an easier and easier-end user version of the finishing table based on the original Mars meter (22). UMARS provides measurement methods for 20 projects, including four objective quality components-participation, function, esthetics, and information quality and one subjective quality component table. Each category includes 3–5 separate problems. Each question has five possible answers, five represents “excellent,” one means “inadequate.” Add the numbers of the questions in each component table and get the average value. The table is the same as the Mars of the Locker five-point scoring method. The score is 1–5 points. Its content validity and reliability review is applied and practiced by experts and is recognized by relevant researchers.

User’s subjective satisfaction is an important part of evaluating the MHEALTH APP. This study uses a semi-structured interview user’s return experience to conduct satisfaction surveys. The interview guide is driven by the existing knowledge in this field. Refer to the research of Lodhia and other research to formulate the interview content, including patient satisfaction, doctors’ effectiveness, and multi-center experience (23, 24). The interview involves the initial issues of openness and targeted follow-up issues including the direct impression of participants to WANHA (Table 1).

**Table 1:** Examples from the semi-structured interview guide.

Sample questions of patient satisfaction	Sample questions of doctor effectiveness
What is your immediate impression of this WeChat applet? Is it esthetically pleasing?	What is your immediate impression of this WeChat applet? Is it esthetically pleasing?
How would you rate the Intelligent Assessment System (is it relevant, easy to use and adequate)?	How would you rate the Intelligent Assessment System (is it accurate and convenient and easy to use)?
How would you evaluate the dental knowledge section (is the information relevant and sufficient)?	Can this WeChat improve the effect of nutritional treatment?
Is it possible to integrate this WeChat applet into your daily life? And how?	Can this WeChat applet reduce the amount of repetitive work you have to do?
What are the advantages and disadvantages of this WeChat applet?	What needs does the WeChat applet meet and what needs does it not meet?
Were you able to find the answers to the questions you were looking for?	

**Figure 1:**
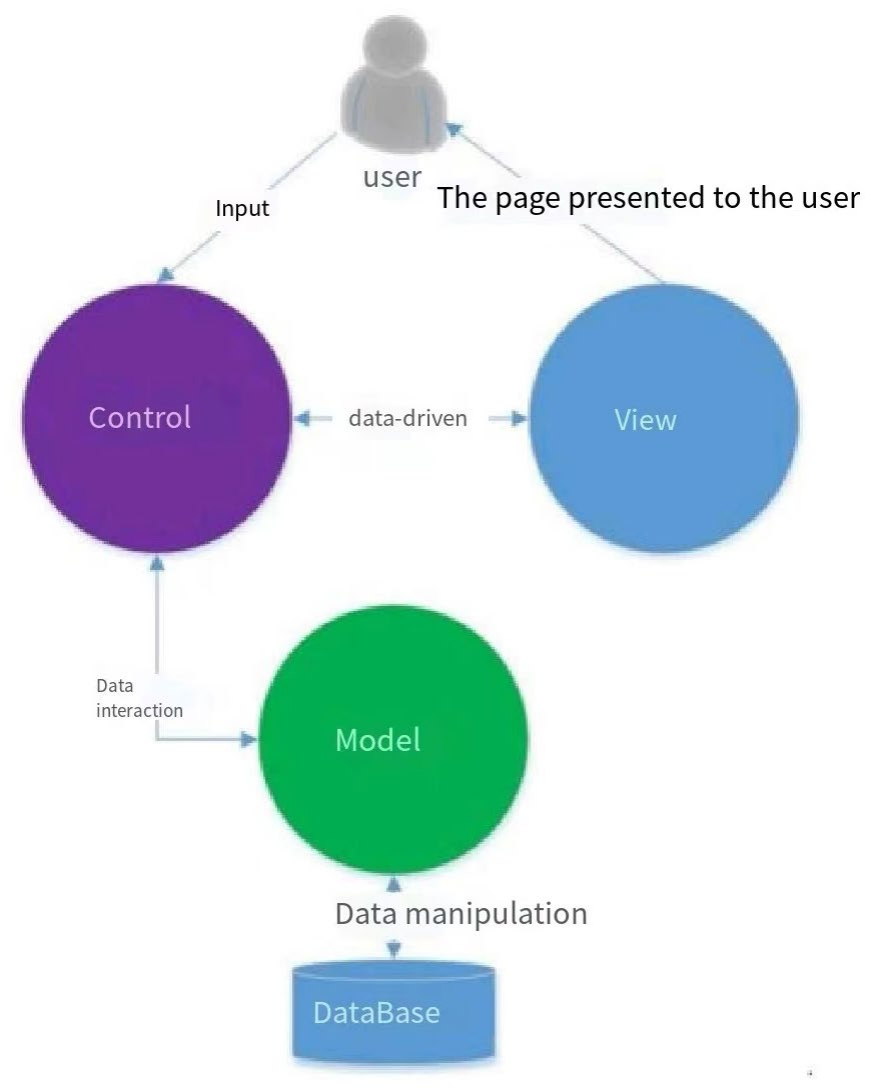
WANHA uses the industry’s popular MVC architecture, which can well ensure the security of the system and data. It has a good user experience, a good application programming interface and later expansion.

**Figure 2:**
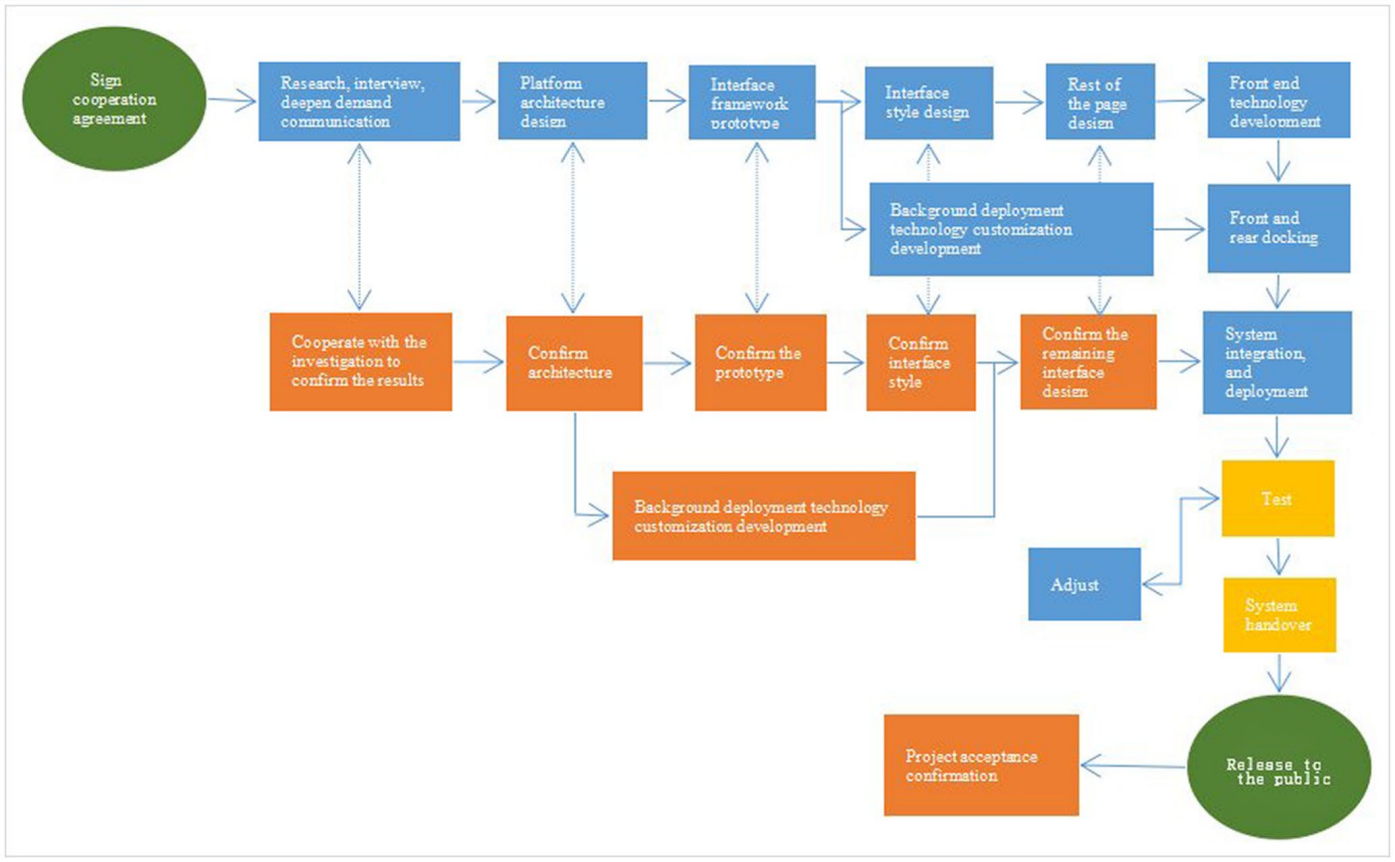
Research and development process.

The WANHA developed by this time uses the industry’s popular Mode View Controller (MVC) architecture to ensure the security of the system and data (25). It has a good user experience, a good application programming interface support and later support and later support and later stage Expansion, the specific system architecture is shown in Figure 1, and the R&D process is shown in Figure 2. The WANHA contains three parts: doctors’ small programs, patient side applets, and web management programs. The medical staff’s applet is filled in the patient’s basic information and the various vital signs of life when admission; the patient’s applet includes NRS2002 (which can be filled in multiple times) and ERAS mission. ERAS knowledge is provided by the consensus or latest related reports involved in the public account of the undergraduate office (26). Publicity column pictures or animations are provided by the undergraduate medical workers or computer scientists (27). The function of the web page is the details of all entry information, the extraction of the patient’s abnormal information, and the general list of each risk screening evaluation (28–30).

### 2.3. Research methods

A multidisciplinary research team selected patients who underwent malignant tumor surgery and medical workers between April 2022 and November 2022. All participants used WANHA to support the treatment of patients with nutritional risks, compared postoperative complications, incidence of symptoms, and postoperative hospitalization. The inclusion criteria were as follows: ability to access the Internet via mobile phone data or Wi-Fi with the help of a relative, ASA classification ≤ III, patients undergoing vocal tumor surgery aged 40–70 years (31–33). Medical personnel involved in the development of WANHA and patients unable to use smartphones to complete nutritional risk screening were excluded from the study. At the end of the study period, the research team adopted a layered random sampling method to select patients. Together with 20 medical workers, a system availability table (SUS) and the mobile application quality rating table was used.

Participants in this research were required to be COVID-19 negative (34, 35), and adhere to a screening protocol which involved providing a COVID-19 negative test report 48 prior to any procedure and wearing a face-shield and mask. Further, patients were required to participate in weekly nucleic acid detection, questionnaire surveys, and follow-up via WeChat video calls in December 2022.

Data analysis was performed using the SPSS statistical software [Version 27, SPSS, Inc. software (Chicago, Illinois, United States)]. The quantitative Licter meter data are presented as mean ± SD. The interview analysis framework used the Rich and Spencer fixed framework. This method is suitable for the existing knowledge and new unpredictable themes related to the instant experience when using the applet (36), and includes five parts: (1) familiar data, (2) determining the theme framework, (3) the theme index, (4) drawing the theme into a layered framework, and (5) mapping and interpretation of themes.

## 3. Results

### 3.1. The WeChat Applet of nutrition and health assessment

**Figure 3:**
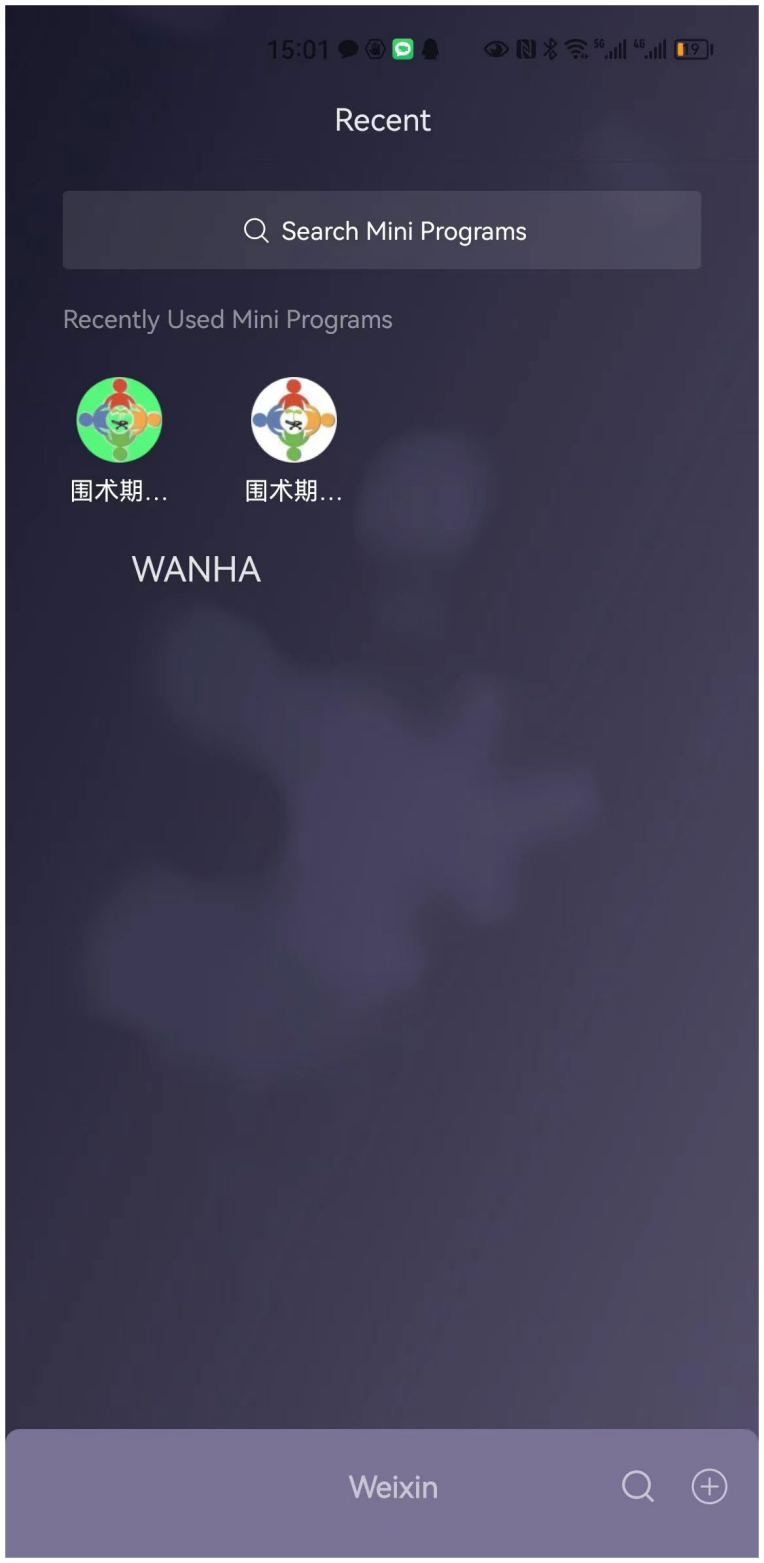
WANHA is a WeChat Mini Program, which contains the side of the medical staff and the patient.

WeChat Applet of Nutrition and Health Assessment is a WeChat mini-program. Anyone can start using the Applet to open the interface at any time on WeChat. Patients and medical staff can scroll the two entry ports of WANHA on the WeChat main screen, namely the patient side (perioperative evaluation) and the medical staff’s side (perioperative evaluation management), and use it (Figure 3). There is very little memory, and it is convenient to use.

**Figure 4:**
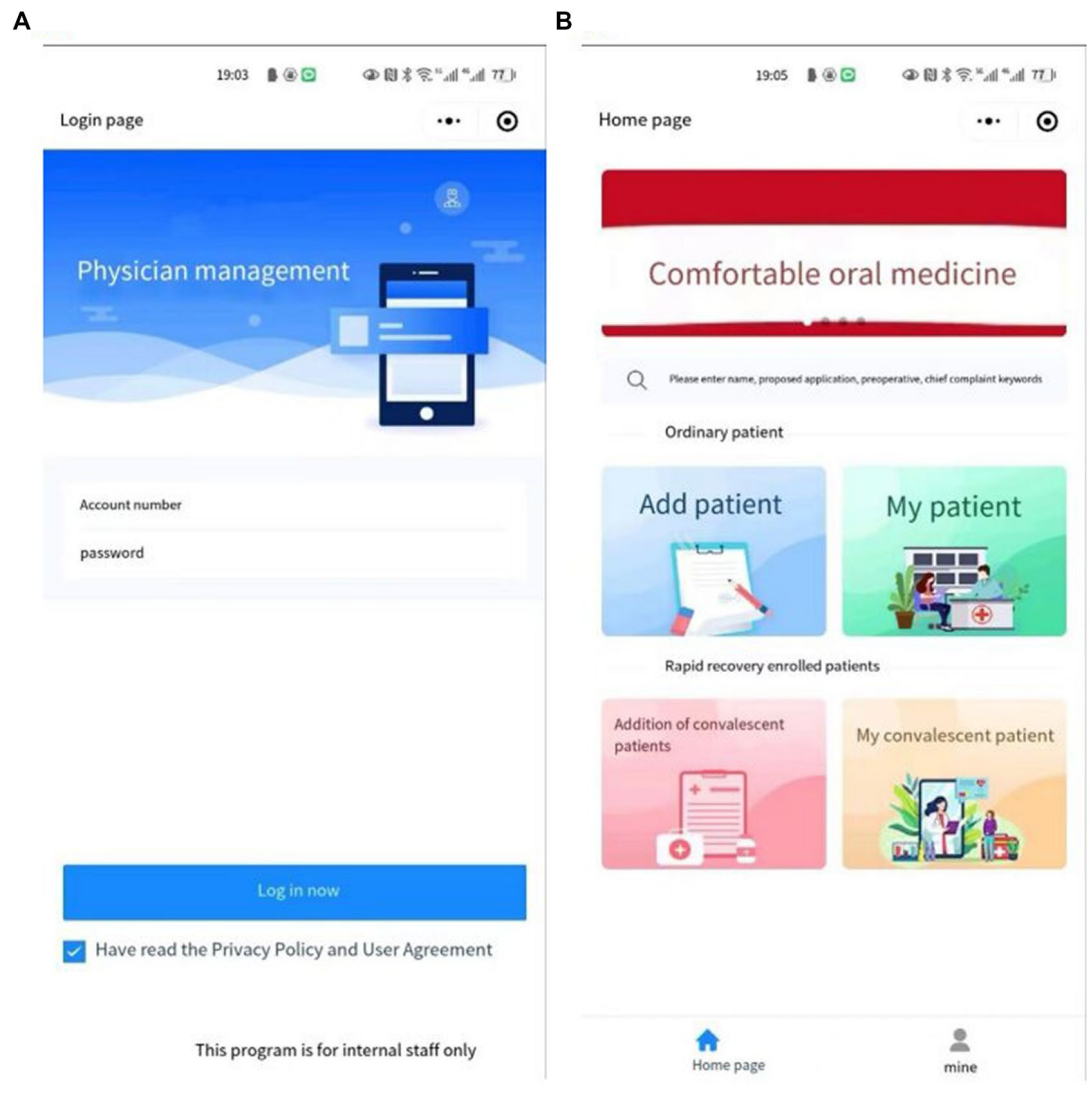
Account password login interface **(A)**, add the patient’s information interface **(B)**.

**Figure 5:**
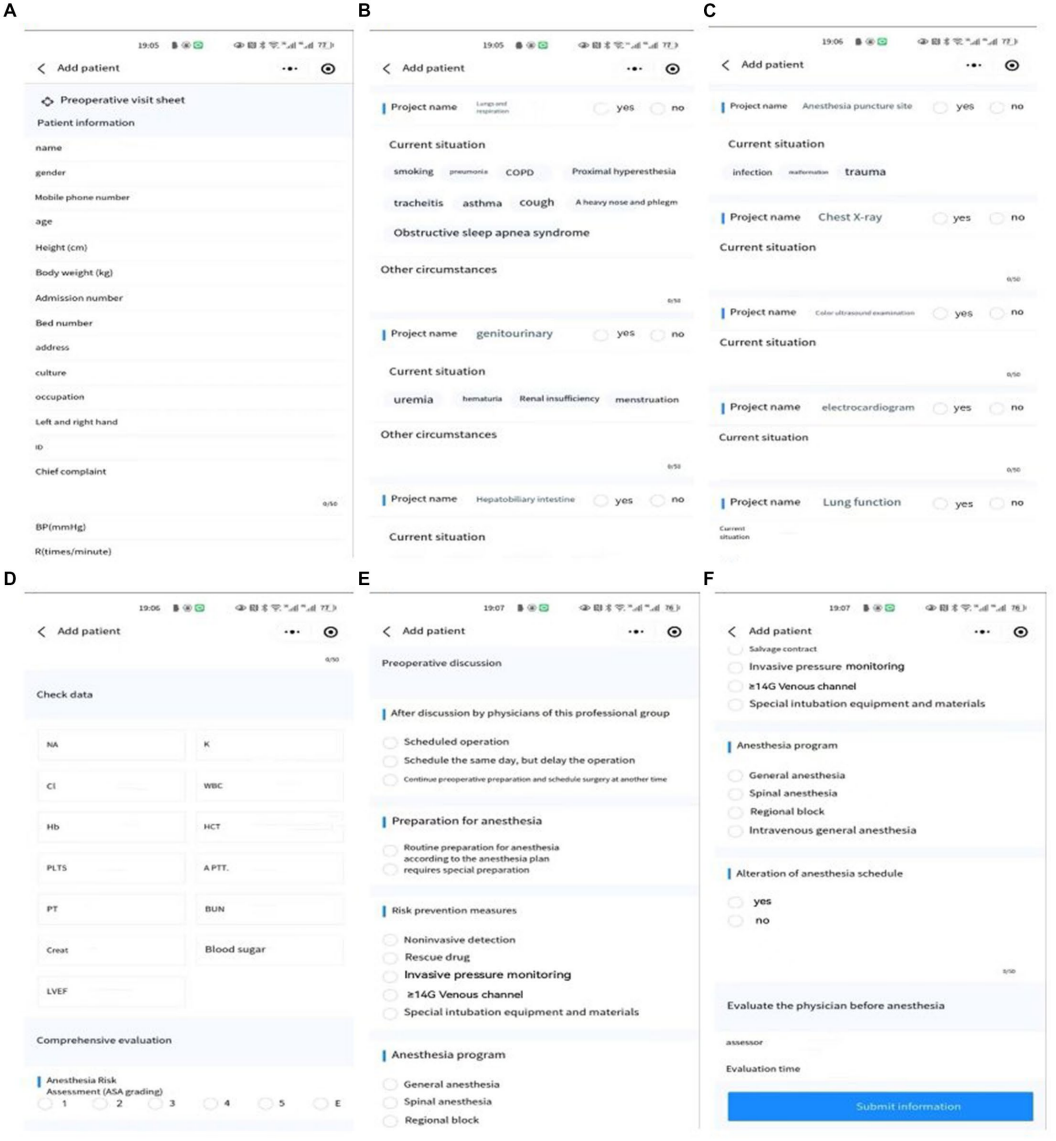
The patient’s basic information, including the general information of the patient **(A)**, history and signs of various systems **(B)**, auxiliary inspection **(C)**, laboratory inspection **(D)**, preoperative **(E)**, and anesthesia plans **(F)**.

Medical staff can log into their password protected user account (Figure 4A) in the patient interface (Figure 4B), and then directly enter the patient’s basic information (Figure 5), including the general information of the patient (Figure 5A), history and signs of various systems (Figure 5B), auxiliary inspection (Figure 5C), laboratory inspection (Figure 5D), preoperative (Figure 5E), and anesthesia plans (Figure 5F).

**Figure 6:**
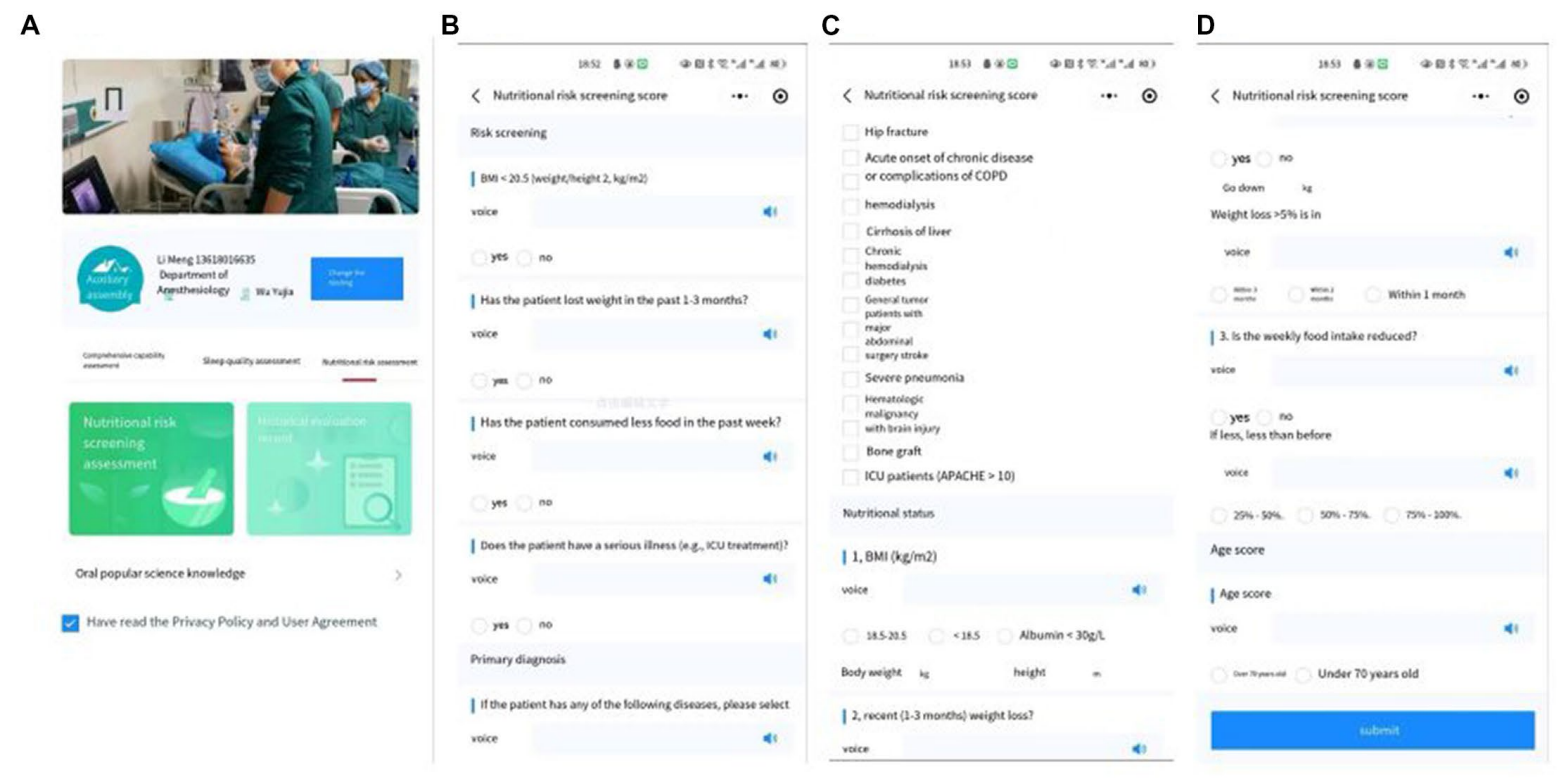
NRS2002 login interface **(A)**. The specific content of NRS2002 **(B–D)**.

**Figure 7:**
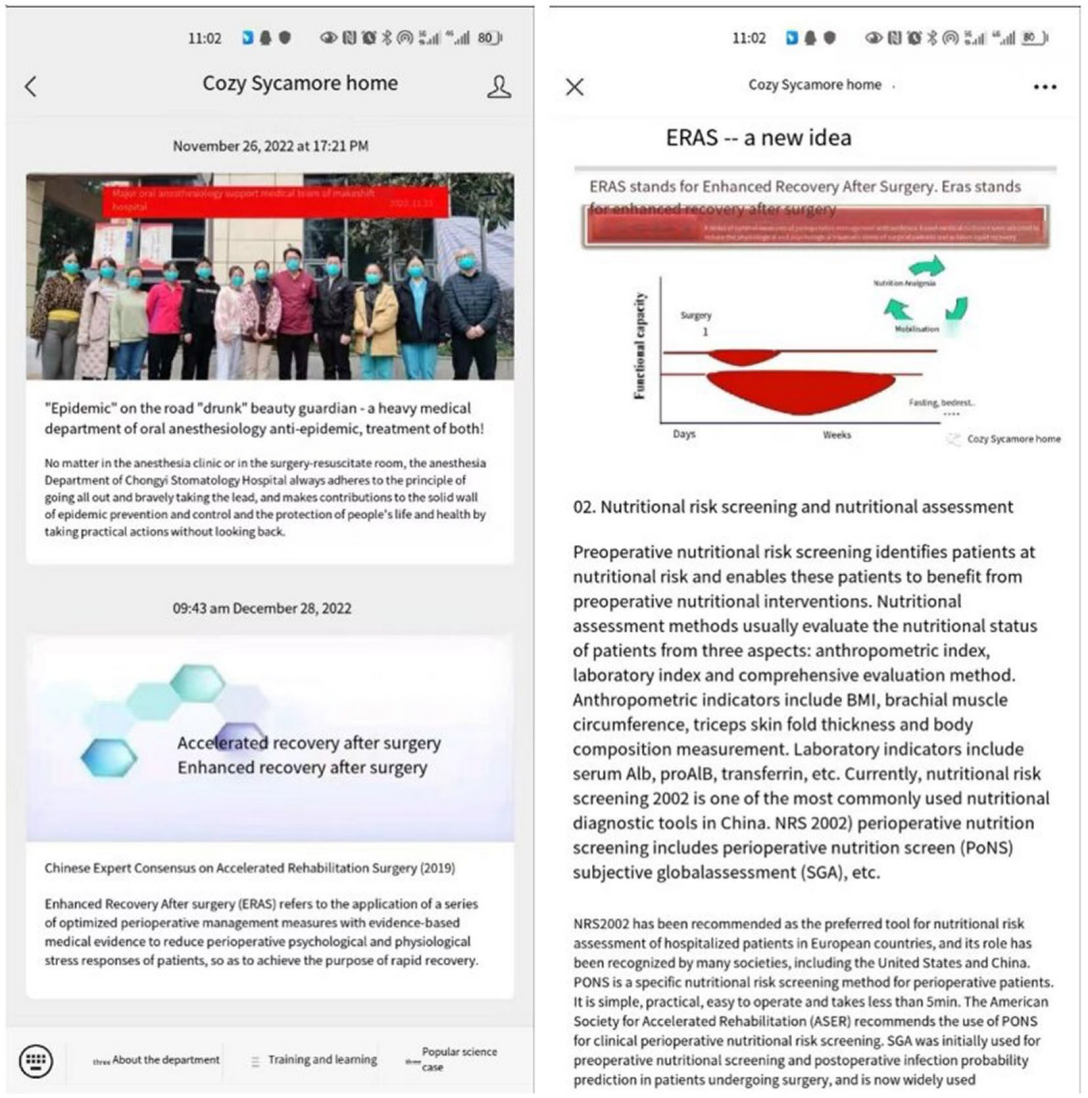
Popular science knowledge related to ERAS.

Patients can log into their side (Figure 6) and fill in the NRS2002 (Figures 6A–C) directly with their name and telephone number. The straightforward design of this app allows it to reach a wider audience. Patients can also improve their awareness through ERAS-related knowledge (Figure 7) and cooperate better with medical workers to carry out ERAS work. After the patient filled out the basic data, the WAMHA system automatically scored it. Both the medical staff and patients can view the general and specific scores of their respective ports.

**Figure 8:**
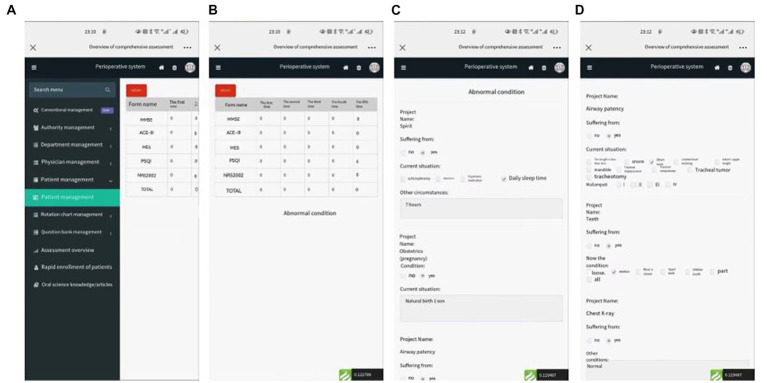
Patient’s nutritional evaluation score table **(A,B)** and nutritional assessment scale **(C,D)**.

Medical staff can check the patient’s details, abnormal basic information, and final score using WANHA’s web management procedure. After a comprehensive analysis, the patient was provided with proper suggestions for nutritional rehabilitation. The WANHA system can show multiple times (at the hospital, 7 days after surgery, and 1 month after surgery) NRS2002 points and abnormal information (Figure 8) enable the medical staff to track and control the continuous nutrition recovery of patients. This can help patients quickly restore their ideal nutritional state and achieve the best effect of ERAS.

### 3.2. Basic information and postoperative situation of participants

This study was jointly involved with multidisciplinary research. A total of 228 patients with malignant tumors were included, of whom 20 were medical workers. All patients used WANHA for nutritional risk screening when they were admitted to the hospital, 7 days after surgery, and 1 month after surgery. After excluding patients who did not participate for personal reasons, 192 patients were eventually included in the study. Among them, 58.8% (113∕192) and 41.2% (79∕192) were men and women with malignant tumors, respectively. Among the 20 medical staff 45% (9∕20) were men and 55% (11∕20) were women. Nutritional risk screening showed that 44.3% (85∕192) of patients had nutritional risks at admission (Table 2). Nutritional support was provided to 85 patients in the perioperative period. Compared with patients who have not been treated during the perioperative period, the incidence of postoperative complications (22.4%∕47.7) and the average hospitalization time after surgery (8∕14.8) have decreased significantly (Table 3). A total of 141 patients (73.4%) who had nutritional risks 7 days after surgery also continued to receive nutritional support treatment and continued the MANHA tracking survey. We found that 83 of the 192 patients who participated in the study had nutritional risks 1 month after the operation and the basic and preoperative rates were the same. It can be seen that MANHA has achieved good results in the perioperative and long-term management.

**Table 2:** Demographic data of participants.

Department	Number of patients (M/F)	Number of medical staff (M/F)	Age of patients	Number of nutritional risk on admission	Number of nutritional risk at 7 days after surgery	Number of nutritional risk at 1 month after surgery
Stomatology	50 (38/12)	5 (3/2)	40–70	19	34	21
Gastrointestinal	38 (23/15)	4 (2/2)	50–68	21	32	19
Hepatobiliary	43 (27/16)	3 (2/1)	40–65	20	31	18
Gynecology	25 (0/25)	3 (0/3)	40–60	13	19	11
Urinary	36 (25/11)	5 (2/3)	60–70	12	25	14
Total	192 (113/79)	20 (9/11)	40–70	85	141	83

**Table 3:** Recovery situation of participants.

Department	Nutritional support group (*n* = 85)		No nutritional support group(*n* = 107)	
	Postoperative complications (%)	Average stay of postoperative (d)	Postoperative complications (M/F)	Average stay of postoperative (d)
Stomatology	42.1 (8/19)	10	54.8 (15/31)	15
Gastrointestinal	33.3 (7/21)	5	47.1 (8/17)	11
Hepatobiliary	40.0 (8/20)	7	52.2 (12/23)	18
Gynecology	30.7 (4/13)	10	41.7 (5/12)	17
Urinary	33.3 (4/12)	8	45.8 (11/24)	13
Total	22.4 (31/85)	8	47.7 (51/107)	14.8

### 3.3. Availability assessment

At the end of the research period, 45 patients were selected from layered random samples [Random sampling, also known as type random sampling. It is to first divide the overall units into various types (or layers) according to certain standards; then according to the ratio of the number of units to the overall unit, the number of units; finally, the samples are extracted from various types in accordance with the principle of random principles], and the SUS was used for follow-up interviews. In the SUS meter, each problem is two points into a neutral value that maintain a neutral attitude. In this study, the average score for each problem was greater than two points (Table 4). WANHA’s SUS score was 72.77 points, indicating good availability and satisfaction.

**Table 4:** The usability assessment of the mobile system (*N* = 65).

	Mean score	SD
1. I think that I would like to use this system frequently	2.40	0.79
2. I found the system unnecessarily complex	3.20	1.24
3. I thought the system was easy to use	2.20	0.87
4. I think that I would need the support of a technical person to be able to use this system	3.40	1.00
5. I found the various functions in this system were well integrated	2.40	0.77
6. I thought there was too much inconsistency in this system	3.60	1.01
7. I would imagine that most people would learn to use this system very quickly	2.60	0.66
8. I found the system very cumbersome to use	3.80	1.08
9. I felt very confifident using the system	2.20	0.77
10. I needed to learn a lot of things before I could get going with this system	3.31	1.00
The overall value of SUS	72.77	

### 3.4. Quality rating

A total of 45 patients sampled using the UMAR results (Table 5) showed that the objective quality of WANHA was divided into (3.39 ± 0.91), and the highest score contained in the four components was the functional component table (3.70 ± 0.85), followed by the information component table (3.56 ± 0.95), and the average score of the participation component table (2.82 ± 0.86) was the lowest. The esthetic component table (3.49 ± 0.98) and WANHA subjective mass component table (3.49 ± 0.92) were not significantly different.

**Table 5:** The quality assessment of the mobile system (*N* = 65).

Subscale/item		Mean score	SD
Engagement		2.82	0.86
1	Entertainment	1.75	0.71
2	Interest	2.52	0.94
3	Customization	3.12	0.98
4	Interactivity	3.74	0.91
5	Target group	2.95	0.78
Functionality		3.70	0.85
6	Performance	3.72	0.86
7	Ease of use	3.50	0.83
8	Navigation	4.02	0.82
9	Gestural design	3.54	0.87
Esthetics		3.49	0.98
10	Layout	3.55	0.94
11	Graphics	3.49	1.00
12	Visual appeal	3.42	1.00
Information		3.56	0.95
13	Quality of information	3.77	0.91
14	Quantity of information	3.54	1.03
15	Visual information	2.95	1.11
16	Credibility of source	3.98	0.76
Total uMARSa		3.39	0.91
Subjective items		3.49	0.92
17	Would you recommend	4.22	0.76
18	How many times	3.28	1.10
19	How many times	2.77	1.00
20	Overall (star) rating	3.68	0.83

### 3.5. Semi-structured interview

Three of the 45 randomly selected patients died of illness, and six were unable to complete the interview for personal reasons. Two participants refused to participate in the interviews. Finally, 54 participants (34 patients and 20 medical personnel) of different ages and sexes, were randomly mixed in the department, and included in the follow-up interview (Table 6).

**Table 6:** Themes and sub-themes of interview.

Patient satisfaction	Doctor effectiveness	Patient advice	Doctor advice
Increase confidence (28/34)	Remote diagnosis, Preparation of special cases	Add treatment opinion items (23/34)	Add treatment opinion items (9/20)
Increase doctor-patient communication and interaction (22/34)	Obtain more comprehensive data and reduce repetitive work, risk (15/20)	Add nutrition and health tips (16/34)	Upload the test results related to nutrition (10/20)
Grasp the status of myself (19/34)	Optimize and improve treatment		
Give prompt feedback (25/34)			

### 3.6. Patient satisfaction

A reduced diet and weight loss are common symptoms of all malignant tumors. According to the nutritional risk screening in this study, approximately half of the patients (44.3%) needed nutritional support at the time of admission, and the nutritional status 7 days after surgery was even more worrying (73.4%). Almost all of the patients who were interviewed had concerns whether their condition could bear surgery and postoperative recovery. WANHA contains nutritional risk screening, and some popular scientific knowledge (including basic common sense and latest progress) has certain authority and accuracy. This study included patients who underwent WANHA screening. The results show that there are fewer postoperative complications in patients who have received nutritional treatment before surgery, who can basically be discharged within 7 days after surgery, and the recovery of 1 month after surgery is better. 82% of patients believe that the perioperative period can be performed through nutritional knowledge and self -risk cognition, and improving their own conditions through intervention can reduce the risk of surgery, eliminate tension and anxiety, increase the success of surgery, and quickly recover.

“Recently, I felt that I was very thin, and my body was much worse. The doctor said that the surgery was relatively extensive, so I was afraid that my body would not bear it. After using the applet, I felt a little confident.” Patient number 10, male, aged 61 years shared (paraphrased).

“I usually feel good. After I got sick, I worried about this. I am afraid that my body is poor and the risk of surgery is high. Anyway, I just do not dare face it. The family said that I was too nervous and I knew that my body was okay. I also saw various situations that helped me recover quickly. The pressure was much lower, and my mood relaxed.” Patient number 21, male, aged 54 years (paraphrased).

### 3.7. Increased doctor-patient communication and interaction

WeChat Applet of Nutrition and Health Assessment can increase communication and enhance interactions between doctors and patients. Of the patients who recognized this view, 65% said that they could clearly perceive the attention and intention of the medical staff during the entire medical treatment process. At the same time, they were observed to cooperate better with the treatment. Both medical experience and treatment effects obviously improved the relationship between doctors and patients.

The following patients shared their opinions on the doctor-patient communications and interactions.

“When we were hospitalized in the past, we passively accepted doctors’ opinions. We did not have the right to speak if we did not understand professional knowledge, as if we were saying nothing. The situation that knows what it does not know, so that we have a certain resistance to various medical methods. Currently, there are more channels for understanding, and the suggestions of doctors can communicate well, communicate, understand, and cooperate with various inspections and treatments.” Patient number 16, male, aged 42 years (paraphrased).

“*I do not feel afraid of staying in the hospital. I can also say something about the condition. The knowledge of the app is unclear. Doctors or nurses can answer well and feel good*.” Patient number 3, female, aged 68 years (paraphrased).

“*Hospital nurses are very busy and do not need to find them frequently when they learn it. It feels like I went less, they came more than they came, and everyone got along more harmoniously*.” Patient number 22, Female, aged 47 years (paraphrased).

### 3.8. Self-status

WeChat Applet of Nutrition and Health Assessment relies on smartphones that are convenient and fast. In the interviews, 19 patients (56%) felt that the use of the procedure was beneficial to themselves. This is because they could evaluate and master their nutritional health status at any time and seek medical treatment when necessary.

Patients’ opinions on evaluating their self-status were as follows:

“*It is difficult to see a doctor. Many people experience queuing for one hour and five minutes to see a doctor. However, your body does not allow you to go. Similar to gastrointestinal tumors, recovery after surgery depends on the nutritional state. With this procedure, I can control it at any time to reduce the number of unnecessary medical treatments, save time, and reduce the workload of the doctor*.” Patient, number 9, male, aged 51 years (paraphrased).

“*You can see your own situation when you pick it up. Even if you are older than me, you cannot use a smartphone, but your family can help it, which is very convenient*.” Patient number 31, female, aged 70 years (paraphrased).

### 3.9. Receiving prompt feedback

WeChat Applet of Nutrition and Health Assessment can immediately score patients’ nutritional risk screening. Approximately 74% of patients believe that instant results can be reported to the medical staff in time, giving corresponding treatment measures in a timely manner, and shortening the patient waiting or even the entire medical treatment.

The patients’ opinions on giving prompt feedback were as follows:

“*Many hospital examinations need to be performed in advance, and the results will be available for half a day or a day. Even if this kind of filling in the form, doctors need to be scored after filling in. Patients cannot know the results in a short time in most cases. Most patients do not know the results in a short time. With this applet, even if the doctor is busy or there is no time to pay attention, we can check it by ourselves. You can also remember this in a timely manner*.” Patient number 5, male, aged 51 years (paraphrased).

“*None of the methods are too time consuming. This can be saved here, and there can be more elsewhere, which is important in time*.” Patient No. 27, male, aged 64 years (paraphrased).

### 3.10. Doctor effectiveness

#### 3.10.1. Remote diagnosis, preparation of special cases

All medical staff affirmed the WANHA’s remote tracking and evaluation function. They can judge the condition in a timely manner based on the patient’s evaluation results, and prepare and provide corresponding guidance for special or dangerous patients to cope with possible complications or emergencies.

#### 3.10.2. Obtain more comprehensive data and reduce repetitive work risk

WeChat Applet of Nutrition and Health Assessment is portable, allowing for multi-center joint investigation statistics to collect more comprehensive patient data for more accurate and reliable results. Simultaneously, the procedure can also be presented intuitively in the form of the patient’s evaluation in the form of a form, which is convenient to observe the continuous conditions of patients’ nutrition and health and avoid the tediousness of repeated statistics. The statistical risk is caused by negligence. Approximately 75% of the medical staff recognized this perspective.

The medical staff shared their opinions as follows:

“*The score and extraction function of this applet reduces the number of statistical steps and solves the problem of insufficient manpower or time for medical staff. At the same time, it also avoids judgment errors caused by excessive repetition, especially when the number of statistics is large*.” Attending physician, Gastrointestinal surgery, male, aged 35 years.

“*At present, many studies require a large amount of data for support. Multi-centered research solves this problem well, which is conducive to the development of clinical research and makes medical services favorable*.” Deputy chief physician, Gynecology, female, aged 50 years.

#### 3.10.3. Optimize and improve treatment

According to the interviews with medical staff from multiple departments, instant scores can make pre- and post-surgery treatment more timely and comprehensive, leading to better patient optimization and improved treatment outcomes.

### 3.11. Proposal of patients and medical staff

According to the interviews, 68% of patients and 45% of medical staff believed that existing applets should increase feedback on treatment measures. That is, the doctor in charge should give appropriate treatment suggestions in combination with the nutritional risk scores and basic signs of each patient in the procedure, and timely feedback to the patient to avoid other reasons (such as poor communication with the long-term doctors and patients and timing of delayed treatment).

WeChat Applet of Nutrition and Health Assessment users were ordinary people. Therefore, 47% of patients believed that science common sense could be more lifelike and more recent. Thus, adding healthy nutritional tips is a way to better serve the public.

Half of the medical staff suggested adding a passage of inspection results related to nutritional health to improve the program. Consequently, doctors can provide more accurate judgments and treatment plans according to more comprehensive information in the program to promote the establishment of a harmonious medical environment.

## 4. Discussion

With the emergence of mobile programs, various medical care applications have emerged. However, the accuracy and reliability of these programs are questionable. In some studies, the application rate for expert participation and medical evidence was as low as 9% (37). How to better combine the combination of ERAS and MHEALTH APP and promote the improvement of medical service quality is a gap that needs to be addressed urgently (38). In this study, MANHA SUS scoring is 72.77. Multiple studies have shown that an average score of SUS > 68 points indicates that the product’s availability is good, and a better score is between 70 and 80 points (39, 40). In the quality rating, the scores of objective qualities (3.39 ± 0.91) and subjective mass (3.49 ± 0.92) are both acceptable and good, indicating that all participants affirm the quality of the program. However, patients and medical staff also agreed that there are room for improvement, especially in the five problems involved in the participation component table (2.82 ± 0.86).

At present, applications that can be used in the market can be divided into two categories: B2C mode for patients and hospital-oriented B2B mode according to target users (41): (1) Patient version: When patients need professional medical staff, they remind them of (14, 42, 43). (2) The clinical doctor version can be used to optimize medical conditions or it can be an auxiliary pathological diagnosis (44–46). The mobile nutritional health management platform based on WeChat mini programs and artificial intelligence based on the design and development of this study moves the entire management system into the cloud database, establishes a logical relationship through programming, and generates intelligent evaluation results. It can help clinical medical workers improve the systematic management of patients.

The semi-structured interview results showed that most patients and medical staff had high acceptance and recognition of MANHA, especially in terms of long-term tracking and diagnosis, timely feedback, improvement of medical and patient relationships, and improvement of treatment effects. In contrast, it depends on the characteristics of WeChat mini-programs: no installation, tentacles can be available after use, and no need to uninstall it, which greatly simplifies complex operations such as registration and login, and improves the user’s use efficiency (47). In addition, the content of the program is reasonable and requires a short time. Nutrition screening can be performed at all times, and the problem of inconsistent information between doctors and patients can be solved. Most participants provided key suggestions on the shortcomings of MANHA, which were mainly reflected in the interaction between medical staff and patients. The continuous improvement in the later period can consider most of the technologies used in the existing research facing the B2C mode application of patients, such as WeChat mini-program that to solve the information occlusion between medical staff and patients (48), poor communication between medical staff and patients, and poor communication between medical staff and patients. WeChat applets promote the exchange of information between medical staff and patients (49). In the future, we will continue to use WeChat Mini Program to formulate more ERAS intervention measures to better serve the majority of patients and medical staff. After the WeChat Mini Program in this research is promoted in the later period, we will use this program to build an ERAS big data sharing platform and establish a multi -central resource sharing.

## 5. Conclusion

In this study, we designed a mobile application based on the WeChat mini program for nutritional health management. The system includes patient-side applications, doctor-side applications, and network management programs. MANHA was designed and developed by multi-center, multi-field, and multi-character systems, and its availability, quality, and satisfaction have been reliable. Both doctors and patients can obtain information related to patients through this procedure, including nutritional risks, nutritional targets, ERAS-related information, and abnormal signs of data, which also provides a new way for the rapid development of ERAS. In general, WANHA is an intelligent and convenient nutritional health management application that helps the safety and efficiency of ERAS and improves the relationship between doctors and patients and the treatment effect. Based on the existing feedback, despite its popularity, WANHA still has room for improvement. Such as the corresponding intervention after evaluation, and the method of treatment for the category of patients’ own disease.

## Data availability statement

The original contributions presented in the study are included in the article/supplementary material, further inquiries can be directed to the corresponding author.

## Author contributions

YW, XW, and FG participated in the study concept and design and analysis and interpretation of the data. XW and JL collected the data and drafted the manuscript. YW and XW critically revised the manuscript. All authors contributed to the article and approved the submitted version.

## Funding

This trial was supported by the CSA Clinical Research Fund (CSA-A2021-05).

## Conflict of interest

The authors declare that the research was conducted in the absence of any commercial or financial relationships that could be construed as a potential conflict of interest.

## Publisher’s note

All claims expressed in this article are solely those of the authors and do not necessarily represent those of their affiliated organizations, or those of the publisher, the editors and the reviewers. Any product that may be evaluated in this article, or claim that may be made by its manufacturer, is not guaranteed or endorsed by the publisher.
